# Supplementary material for: Scalable MoS2/Si Photodiode Arrays From Roll‐to‐Roll Mechanical Exfoliation
Source: Adv Sci (Weinh). 2026 Jul 27:e76787. Online ahead of print. doi: 10.1002/advs.76787 (PMC13403370; doi:10.1002/advs.76787)
Supplement: Supplementary file 1 — Supporting File: advs76787‐sup‐0001‐SuppMat.pdf. [file ADVS-9999-e76787-s001.pdf]

Supporting Information:

Scalable MoS_2/Si Photodiode Arrays from Roll-to-Roll Mechanical Exfoliation

Yigit Sozen[1], Thomas Pucher[1], Andres Castellanos-Gomez[1]

[1]2D Foundry research group. Instituto de Ciencia de Materiales de Madrid (ICMM-CSIC), Madrid, E-28049, Spain.

*corresponding authors yigit.sozen@csic.es, andres.castellanos@csic.es

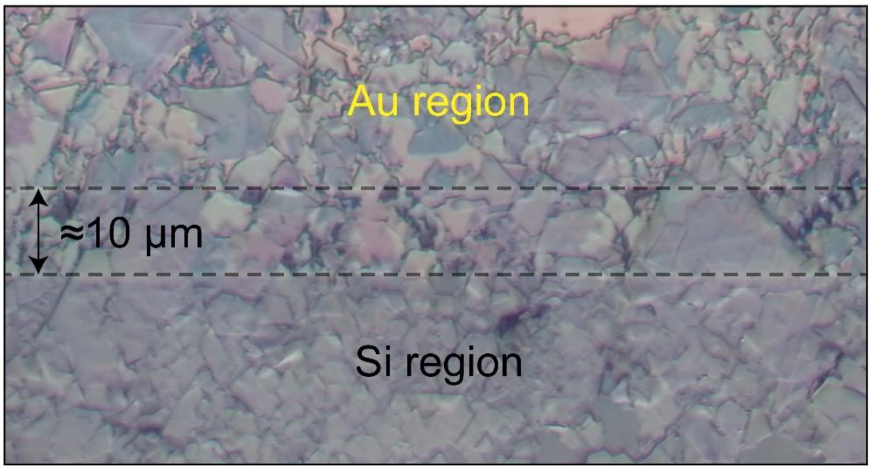

Figure S1. Representative channel area of a pixel covered with MoS_2 flakes. Optical microscope image showing a representative portion of the channel area of a pixel. The Au electrode region is located on the insulating SiO_2 layer and is laterally separated from the exposed Si window by an approximately 10 µm SiO_2-covered gap. The transferred MoS_2 flakes bridge this gap, providing electrical connection between the Au electrode and the exposed Si region, while the MoS_2/Si heterojunction is formed only at the exposed Si window.

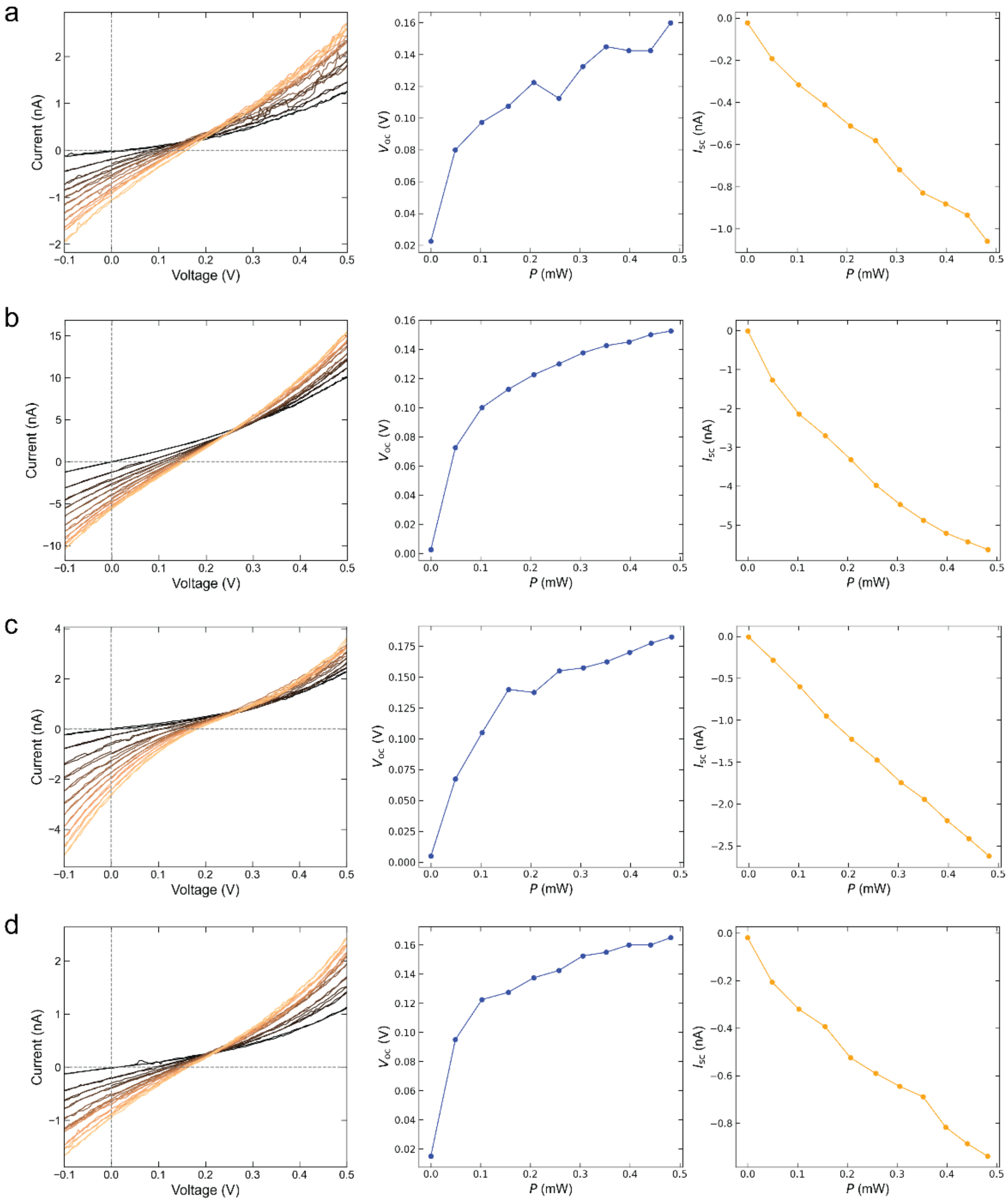

Figure S2. Optoelectronic characterization of different MoS_2/Si photodiodes. (a–d) Current–voltage (IV) characteristics measured under increasing illumination power (P), and the extracted open-circuit voltage (V_{OC}) and short-circuit current (I_{SC}) values plotted as a function of P, shown in the left, middle, and right panels, respectively.

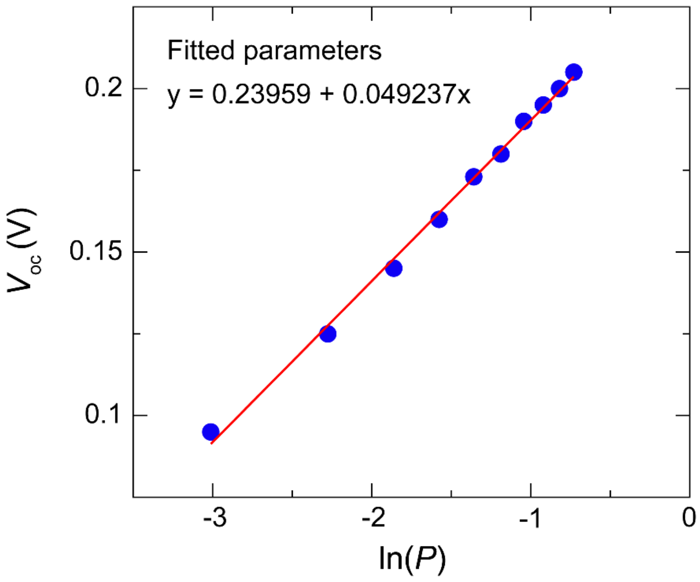

Figure S3. Extraction of the diode ideality factor. V_{OC} versus $\ln(P)$ with corresponding linear fit (solid red line) used for extraction of the ideality factor.

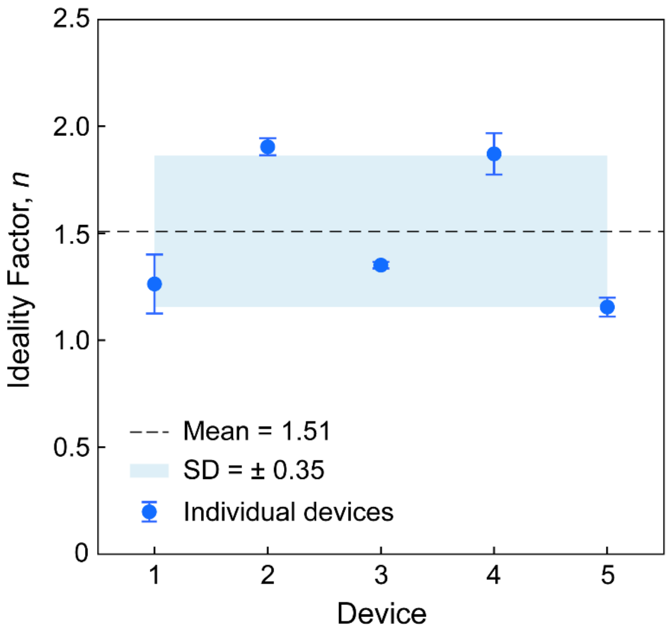

Figure S4. Distribution of ideality factors across MoS_2/Si photodiodes. Plot shows the ideality factors extracted from five MoS_2/Si pixels in linear pixel array. The data points represent the ideality factor values obtained from the linear fit of the V_{OC} versus $\ln(P)$ characteristics for each pixel. The vertical error bars indicate the uncertainty of the extracted ideality factor, calculated from the standard error of the fitted slope. The dashed horizontal line represents the average ideality factor, $n_{\text{avg}} = 1.51$, while the shaded region corresponds to the device-to-device standard deviation, $n_{\text{avg}} \pm \text{SD}$, with $\text{SD} = 0.35$.

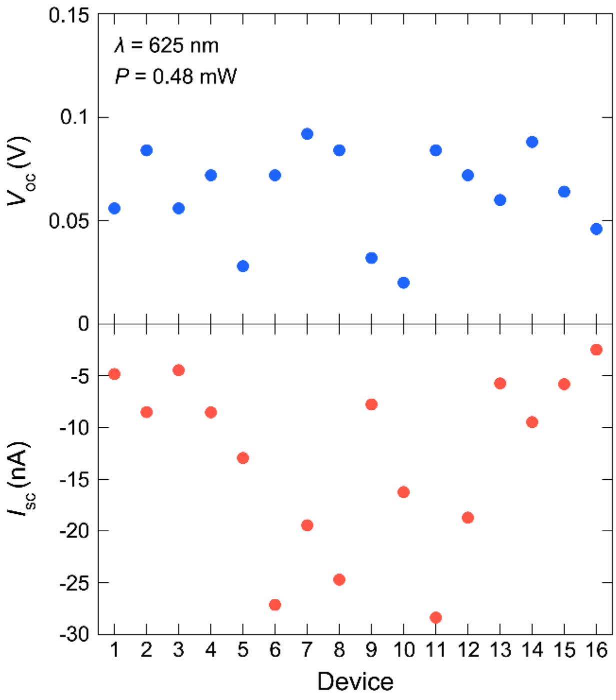

Figure S5. Photovoltaic parameters from the 4×4 MoS_2/Si photodiode array. The plot shows the extracted V_{OC} and I_{SC} values for all 16 pixels in the 4×4 photodiode array.
